# Predicting the risk of 7‐day readmission in late preterm infants in California: A population‐based cohort study

**DOI:** 10.1002/hsr2.994

**Published:** 2023-01-02

**Authors:** Ribka Amsalu, Scott P. Oltman, Melissa M. Medvedev, Rebecca J. Baer, Elizabeth E. Rogers, Stephen C. Shiboski, Laura Jelliffe‐Pawlowski

**Affiliations:** ^1^ California Preterm Birth Initiative University of California San Francisco San Francisco California USA; ^2^ Department of Epidemiology & Biostatistics University of California San Francisco San Francisco California USA; ^3^ Department of Pediatrics University of California San Francisco San Francisco California USA; ^4^ London School of Hygiene & Tropical Medicine, Maternal, Adolescent, Reproductive and Child Health Centre London UK; ^5^ Department of Pediatrics University of California San Diego La Jolla California USA

**Keywords:** prediction, preterm, rehospitalization, risk stratification

## Abstract

**Background and aims:**

The American Academy of Pediatrics describes late preterm infants, born at 34 to 36 completed weeks' gestation, as at‐risk for rehospitalization and severe morbidity as compared to term infants. While there are prediction models that focus on specific morbidities, there is limited research on risk prediction for early readmission in late preterm infants. The aim of this study is to derive and validate a model to predict 7‐day readmission.

**Methods:**

This is a population‐based retrospective cohort study of liveborn infants in California between January 2007 to December 2011. Birth certificates, maintained by California Vital Statistics, were linked to a hospital discharge, emergency department, and ambulatory surgery records maintained by the California Office of Statewide Health Planning and Development. Random forest and logistic regression were used to identify maternal and infant variables of importance, test for association, and develop and validate a predictive model. The predictive model was evaluated for discrimination and calibration.

**Results:**

We restricted the sample to healthy late preterm infants (*n* = 122,014), of which 4.1% were readmitted to hospital within 7‐day after birth discharge. The random forest model with 24 variables had better predictive ability than the 8 variable logistic model with c‐statistic of 0.644 (95% confidence interval 0.629, 0.659) in the validation data set and Brier score of 0.0408. The eight predictors of importance length of stay, delivery method, parity, gestational age, birthweight, race/ethnicity, phototherapy at birth hospitalization, and pre‐existing or gestational diabetes were used to drive individual risk scores. The risk stratification had the ability to identify an estimated 19% of infants at greatest risk of readmission.

**Conclusions:**

Our 7‐day readmission predictive model had moderate performance in differentiating at risk late preterm infants. Future studies might benefit from inclusion of more variables and focus on hospital practices that minimize risk.

## 1 INTRODUCTION

Readmission within 30‐day after index admission is used as a quality‐of‐care measure in adult medicine.^1^, ^2^ However, the appropriateness of the 30‐day cutoff for pediatric patients is controversial. Early readmission, defined as readmission within 7‐day from discharge, is preferred to approximate preventability.^3^ In the neonatal period, late preterm infants (LPTs), born at 34 to 36 completed weeks' gestation, are at two‐to‐three‐fold increased risk of readmission after birth as compared to term infants.^4^, ^5^ The majority of these readmissions occur shortly after birth discharge and are primarily due to hyperbilirubinemia, feeding difficulties, infection/sepsis, or respiratory complications.^5^, ^6^, ^7^

Efforts taken to minimize the risk of unplanned early readmission, such as longer length of birth hospitalization, have mixed outcomes;^8^ predischarge bilirubin screening and subthreshold phototherapy during birth hospitalization have shown promise, however, the number needed to treat is large.^9^, ^10^ Differentiating those who are at increased risk of unplanned early readmission following birth hospitalization could potentially inform targeted predischarge care and transition planning. Previous studies have identified factors that may be useful for such differentiation including length of stay at birth hospitalization, gestational age, and predischarge bilirubin screening.^5^, ^10^ To the best of our knowledge, only one study has developed a predictive model for readmission that includes LPTs. Escobar's^11^ 30‐day readmission predictive model for all neonates including LPTs, which includes maternal age, sex, gestational age, Score for Neonatal Acute Physiology‐II (SNAP‐II),^12^ facility of birth, and follow‐up after discharge, had a c‐statistic of 0.66. This study builds on Escobar's predictive model by adding maternal and infant morbidity variables and deriving individual risk scores and risk classification.

## 2 METHODS

### 2.1 Cohort selection

The cohort was drawn from California live births between January 1, 2007, and December 31, 2011. Birth certificates, maintained by California Vital Statistics, were linked to a hospital discharge, emergency department, and ambulatory surgery records maintained by the California Office of Statewide Health Planning and Development. The linkage algorithm included variables such as birth hospital, date of birth, sex, zip code, race/ethnicity, and hospital discharge records that are also recorded on the birth certificates. Of the 3,448,707 infants recorded in the birth certificate file, 91.3% had hospital discharge records linked to both mother and infant. Infants who had a birth admission discharge status indicating a transfer to another hospital were identified as “transferred” and those whose birth admission discharge status indicated death as “died during birth hospitalization.” California birth certificates include a variable that indicates if an infant was admitted to a neonatal intensive care unit (NICU). By excluding LPTs who were transferred, died, admitted to a NICU during birth hospitalization, or had major congenital anomalies,^13^ we aimed to limit the sample to those presumed healthy at birth hospitalization. Information on diagnosis was based on the International Classification of Diseases, 9th Revision, Clinical Modification (ICD‐9) and International Classification of Diseases, 10th Revision, Clinical Modification (ICD‐10).^14^ A complete case analysis was performed, as missing data for predictor variables of interest were minimal (<3%).

### 2.2 Outcome

The outcome of interest, readmission within 7‐day, was defined as any LPTs readmitted to hospital within 7‐day after birth hospitalization.

### 2.3 Predictor variables

Biological plausibility, accuracy in measurement, availability in the database, and reliability of record were criteria applied by the study team to narrow the list of candidate variables from 34 (identified *a priori* based on literature) to 24 (Supporting Information: eTable 1). The candidate variables included maternal and infant characteristics, maternal and infant morbidity, and healthcare payor. Our database had limited infant morbidity variables and the disease conditions, though identified in literature as contributing to readmission in preterm infants, that were available to us were mostly prevalent in early preterm infants.

### 2.4 Statistical analysis

A four‐step approach was used to develop and validate the predictive models. First, the cohort was randomly divided into a training/derivation sample including 80% of infants, and a validation sample including 20% of infants. Random forests, a supervised machine learning technique, was then applied to the derivation sample to rank candidate variables of importance.^15^ Three predictive models were derived: (1) model 1 ‐ a random forest including all (24) candidate variables; (2) model 2‐ a logistic regression model including eight predictor variables, which were selected based on the Gini importance derived from the random forest and statistical significance (*p* value); and (3) model 3 ‐ a 7‐predictor logistic regression model‐based on the 8‐predictor model but excluding race/ethnicity as a variable. We then developed a risk score by assigning points proportional to the *β*‐coefficient values of the eight predictors in model 2, and three risk categories (protective, neutral, and risk) were created.

The predictive model was evaluated using the c‐statistic, performance parameters of sensitivity, specificity, positive predictive value, and negative predictive value, Brier score,^16^ and a calibration plot of predicted versus observed risk of readmission. Individual risk scores were plotted against the predicted risk of readmission to assess performance of the risk classification. Statistical analyses were performed using SAS, version 9.4 (SAS Institute Inc) and R version 4.0.2.^17^

### 2.5 Ethics approval

The study was approved by the Committee for the Protection of Human Subjects of the California Health and Human Services Agency. The manuscript adheres to the Transparent Reporting of a Multivariable Prediction Model for Individual Prognosis or Diagnosis (TRIPOD) statements and explanations.^18^

## 3 RESULTS

The study population was 122,014 LPTs, of which 4.1% (*n* = 5017) were readmitted within 7‐day from birth hospitalization. The derivation (*n* = 97,611) and validation (*n* = 24,403) samples had comparable characteristics including similar readmission (4.1% vs. 4.3%), mean length of stay, gestational age breakdown, maternal morbidity, and infant morbidity (Supporting Information: eTable 2).

Predictor variables with the highest random forest important rankings were all included in the logistic regression. Logistic regression results for the derivation sample revealed that longer length of stay (adjusted odds ratio [aOR] 0.93 [95% confidence interval [CI]: 0.91, 0.95]) and cesarean delivery (aOR 0.73 [95% CI: 0.68, 0.78]) were protective. Assisted vaginal delivery (aOR 1.39 [95% CI: 1.18, 1.64]), primiparity (aOR 1.38 [95% CI: 1.27, 1.49]), and receiving phototherapy (aOR 1.71 [95% CI: 1.54, 1.90]) were risk factors (Table 1).

**Table 1:** Multivariable predictive model of risk of readmission within 7 days after birth hospitalization in late preterm infants (derivation sample, *n* = 97,611)

Predictors	*β* coefficient	Standard error	Adjusted odds ratio (95% CI)	*p* Value
Intercept	−3.7455	0.1281		<0.0001
Birth length of stay in days	−0.0731	0.00848	0.93 (0.91, 0.95)	<0.0001
Delivery method				
Assisted vaginal	0.3304	0.0843	1.39 (1.18, 1.64)	<0.0001
Cesarean section	−0.3157	0.0364	0.73 (0.68, 0.78)	<0.0001
Vaginal	Reference	Reference	Reference	Reference
Phototherapy			1.71 (1.54, 1.90)	<0.0001
Parity				
Para zero	0.319	0.0411	1.38 (1.27, 1.49)	<0.0001
1	−0.00601	0.0445	0.99 (0.91, 1.09)	0.8925
2–4	Reference	Reference	Reference	Reference
≥5	−0.074	0.1051	0.93 (0.76, 1.14)	0.4814
Unknown	0.5264	0.463	1.69 (0.68, 4.20)	0.2555
Gestational Age in Weeks				
34	−0.0844	0.083	0.92 (0.78, 1.08)	0.3093
35	0.219	0.0377	1.25 (1.16, 1.34)	<0.0001
36	Reference	Reference	Reference	Reference
Birthweight (per 100 g)	0.0244	0.00397	1.03 (1.02, 1.03)	<0.0001
Race/ethnicity				
Asian	0.2462	0.0502	1.28 (1.16, 1.41)	<0.0001
Black	−0.5366	0.0935	0.59 (0.49, 0.70)	<0.0001
Hispanic	−0.0574	0.0416	0.94 (0.87, 1.02)	0.1674
White	Reference	Reference	Reference	Reference
Other	0.00749	0.0779	1.01 (0.87, 1.17)	0.9234
Pre‐existing or gestational diabetes	0.1463	0.0426	1.16 (1.07, 1.26)	0.0006

**Figure 1:**
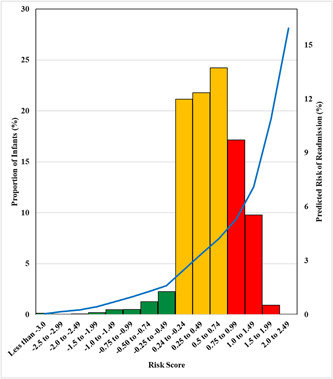
Risk score to predict 7‐day readmission in late preterm infants in California. Predicted risk of readmission plotted against risk scores in the validation sample (*n* = 24,403); bars indicate proportion of late preterm infants by risk score; blue line indicates predicted readmission risk, color codes signify risk category (blue = protective, yellow = neutral, red = at risk).

The random forest had the largest c‐statistic of 0.668 (95% CI: 0.661, 0.676), followed by 0.613 (95% CI: 0.604, 0.622) for the 8‐predictor logistic model (Table 2). The Brier score of the models ranged from 0.0385 to 0.0387. In general, the observed and predicted risks were close to each other, (Figure 1), where predicted risks are within the 95% CI of observed risks for most population deciles and those in the higher risk subgroup for readmission.

**Table 2:** Performance of predictive model for risk of 7‐day readmission in late preterm infants in California in derivation and validation sample

	8‐predictor logistic model		7‐predictor logistic model		Random forests	
	Derivation	Validation	Derivation	Validation	Derivation	Validation
C‐statistic	0.613	0.595	0.604	0.594	0.668	0.644
95% CI	(0.604, 0.622)	(0.577, 0.613)	(0.595, 0.613)	(0.577, 0.612)	(0.661, 0.676)	(0.629, 0.659)
Brier	0.03869	0.04095	0.03873	0.040969	0.03852	0.04081
Sensitivity	57.6%	60.6%	54.2%	62.4%	59.3%	56.6%
95% CI	(56.1%, 59.2%)	(57.6%, 63.6%)	(52.6%, 55.7%)	(59.4%, 65.4%)	(57.7%, 60.8%)	(53.6%, 59.6%)
Specificity	58.9%	53.1%	61.5%	52.0%	64.8%	64.6%
95% CI	(58.6%, 59.2%)	(52.5%, 53.8%)	(61.2%, 61.8%)	(51.4%, 52.7%)	(64.5%, 65.1%)	(64.0%, 65.2%)
Positive predictive value	5.9%	5.5%	6.0%	5.5%	6.7%	6.7%
95% CI	(5.8%, 6.1%)	(5.2%, 5.8%)	(5.8%, 6.1%)	(5.3%, 5.8%)	(6.5%, 6.8%)	(6.4%, 7.1%)
Negative predictive value	96.9%	96.8%	96.8%	96.9%	97.4%	97.1%
95% CI	(96.8%, 97.0%)	(96.5%, 97.0%)	(96.7%, 96.9%)	(96.6%, 97.1%)	(67.3%, 97.5%)	(96.9%, 97.3%)

The validation sample yielded similar model performance with no overfitting, the random forest had c‐statistic of 0.644 (95% CI: 0.629, 0.659) and a Brier score of 0.0408; and the calibration plot indicated performance comparable to the derivation sample (Supporting Information: eFigure 1). The risk scoring and stratification in the validation sample, based on the *β*‐coefficients of the 8‐predictor logistical model (formula in Figure 1), yielded individual risk scores ranging from less than −3.0 to 2.5 and was heavily left‐skewed. An estimated 1% of cohort participants were categorized as having reduced risk (scores of <−0.24); 63% were categorized as having neutral (−0.24 to 0.74), and 19% were considered as having significant risk (≥0.75). Higher risk scores corresponded to higher predicted risk of readmission.

## 4 DISCUSSION

### 4.1 Principal findings

In this large, multicenter, population‐based cohort study, we found a 7‐day readmission rate of 4.1% among LPTs. Our 7‐day readmission predictive model had moderate performance (c‐statistic = 0.644, Brier score = 0.0408) in the validation sample. The risk stratification strategy exhibited some capacity for predicting risk of readmission.

### 4.2 Strength and limitations

Our predictive model is novel due to its focus on LPTs, early readmission, and use of routinely collected variables. Limitations were unavailability of potentially important candidate predictor variables such as breastfeeding, weight loss, bilirubin screening, dehydration, respiratory complications, and sepsis. While several morbidities that are more prevalent in very early preterm infants population were available in the database and have been found to increase risk of readmission, hence included in Table 1, they were not variables of importance in healthy late preterm infant population and were not in the final predictive model. Inability to differentiate planned versus unplanned readmission was also a limitation. Collinearity is often raised as a potential limitation in regression models for prediction. In our study we decided to include both gestational age and birthweight, as there were no large indications of collinearity (based on standard error and variance of inflation factor) and both variables were ranked as important by the random forests.

### 4.3 Interpretation

LPTs, though more vulnerable to morbidity, mortality, and readmission, often receive similar care to term infants at birth hospitalization. The risk classification strategy developed in our study provides a promising start for future predictive studies and the ability to differentiate LPTs at risk of early readmission. LPTs that had short length of stay, born via assisted vaginal birth, to primipara women, and those who have diabetes need extra attention at predischarge care including assessing parental readiness, screening for hyperbilirubinemia, feeding support, and early follow‐up. The protective effect of cesarean delivery is possibly mediated by prolonged birth hospitalization of mother‐baby dyad and management of complications such as temperature instability, feeding difficulties, sepsis, and hyperbilirubinemia predischarge.^7^

Earlier studies have found that infants ≥36 weeks' gestation who had excess weight loss (≥10% of birthweight) tended to have increased outpatient and inpatient health care utilization in the first month of life as compared to those who had <8% of birthweight loss,^19^ similarly dehydration and feeding difficulties are important predictors to early readmission, as is parental readiness.^20^, ^21^, ^22^ Inclusion of these variables to the model might improve performance.

Aside from the debate whether readmission is appropriate or not to measure neonatal quality of care or to compare hospital performances, it is critical that we identify hospital, physician, and parental practices that have minimized risk of readmission, and the extent of preventability of readmissions in the neonatal period.^23^

## 5 CONCLUSIONS

Early readmission after birth is costly, disruptive to the family, and places preterm infants at risk of nosocomial infections. Our 7‐day readmission predictive model had moderate performance in differentiating at risk LPTs. Predischarge care practices and transition plan need to be informed by maternal and infant variables that are protective or risk factors for readmission. Our study provides the basis for future prospective research and predictive models where more clinical variables could be included in model.

## AUTHOR CONTRIBUTIONS

**Ribka Amsalu**: Conceptualization; data curation; methodology; validation; visualization; writing – original draft. **Scott P. Oltman and Rebecca J. Baer**: Conceptualization; data curation; formal analysis; methodology; writing – review & editing. **Melissa M. Medvedev**: Conceptualization; methodology; writing – review & editing. **Elizabeth E. Rogers, Stephen C. Shiboski, and Laura Jelliffe‐Pawlowski**: Conceptualization; methodology; writing – review & editing.

## CONFLICT OF INTEREST

The authors declare no conflict of interest.

## TRANSPARENCY STATEMENT

The lead author Ribka Amsalu affirms that this manuscript is an honest, accurate, and transparent account of the study being reported; that no important aspects of the study have been omitted; and that any discrepancies from the study as planned (and, if relevant, registered) have been explained.

## Supporting information

Supplementary information.

Supplementary information.

Supplementary information.

Supplementary information.

## Data Availability

The source of data is the California Office of Statewide Health Planning and Development and California Vital Statistics.
