# Supplementary material for: Predicting the risk of 7‐day readmission in late preterm infants in California: A population‐based cohort study
Source: Health Sci Rep. 2023 Jan 2;6(1):e994. doi: 10.1002/hsr2.994 (PMC9808150; doi:10.1002/hsr2.994)
Supplement: Supplementary file 1 — Supplementary information. [file HSR2-6-e994-s003.docx]

| **eTable 1: Candidate predictor variables evaluated for predicting risk of 7-day readmission in late preterm infants** |
| --- |
| **Maternal variables** |
| Maternal age |
| Maternal education |
| Race/Ethnicity |
| Parity |
| Prenatal care |
| Perinatal smoking |
| Mode of delivery |
| Any Hypertension |
| Any Diabetes Mellitus |
| Chorioamnionitis |
| **Infant variables** |
| Sex |
| Birthweight |
| Gestational age |
| Small for gestational age |
| **Infant morbidity** |
| Bronchopulmonary dysplasia |
| Necrotizing enterocolitis |
| Respiratory distress syndrome |
| Intraventricular hemorrhage |
| Retinopathy of prematurity |
| Patent ductus arteriosus |
| Periventricular leukomalacia |
| Phototherapy |
| **Health care variables** |
| Length of Stay |
| Payer (insurance) |
