# Supplementary material for: Predicting the risk of 7‐day readmission in late preterm infants in California: A population‐based cohort study
Source: Health Sci Rep. 2023 Jan 2;6(1):e994. doi: 10.1002/hsr2.994 (PMC9808150; doi:10.1002/hsr2.994)
Supplement: Supplementary file 2 — Supplementary information. [file HSR2-6-e994-s001.docx]

| **eTable 2: Characteristics of study participants with readmitted and not readmitted late preterm infants in the derivation and validation sample (n=122,014)** | | | | |
| --- | --- | --- | --- | --- |
|  |  |  |  |  |
| **Variables** | **Derivation sample** | | **Validation sample** | |
|  | No Readmission N(%) | Readmission N(%) | No Readmission N(%) | Readmission N(%) |
|  | 93645 (95.9) | 3966 (4.1) | 23352 (95.7) | 1051 (4.3) |
| Length of stay (days) mean (sd) | 3.4 (4.2) | 2.8 (1.3) | 3.4 (4.5) | 2.8 (1.3) |
| Birthweight (per 100g) mean (sd) | 27.3 (4.4) | 27.8 (4.1) | 27.3 (4.4) | 27.8 (4.2) |
| Gestational age |  |  |  |  |
| 34 | 6109 (6.5) | 176 (4.4) | 1569 (6.7) | 40 (3.8) |
| 35 | 22801 (24.4) | 1097 (27.7) | 5570 (23.9) | 293 (27.9) |
| 36 | 64735 (69.1) | 2693 (67.9) | 16213 (69.4) | 718 (68.3) |
| SGA, yes | 8447 (9.0) | 254 (6.4) | 2052 (8.8) | 72 (6.9) |
| Sex, Female | 44129 (47.1) | 1736 (43.8) | 11026 (47.2) | 443 (42.2) |
| Delivery mode |  |  |  |  |
| Vaginal | 50526 (54.0) | 2495 (62.9) | 12619 (54.0) | 647 (61.6) |
| Assisted vaginal | 2110 (2.2) | 165 (4.2) | 489 (2.1) | 39 (3.7) |
| Cesarean delivery | 41009 (43.8) | 1306 (32.9) | 10244 (43.9) | 365 (34.7) |
| Prenatal care |  |  |  |  |
| Inadequate | 9856 (10.5) | 387 (9.8) | 2422 (10.4) | 109 (10.4) |
| Intermediate | 6824 (7.3) | 327 (8.3) | 1700 (7.3) | 81 (7.7) |
| Adequate | 27605 (29.5) | 1270 (32.0) | 6979 (29.9) | 323 (30.7) |
| Adequate Plus | 46632 (49.8) | 1894 (47.8) | 11576 (49.6) | 109 (10.4) |
| Unknown | 2728 (2.9) | 88 (2.2) | 675 (2.9) | 29 (2.8) |
| Payment type |  |  |  |  |
| Medi-Cal/public | 43411 (46.4) | 1867 (47.1) | 10882 (46.6) | 528 (50.2) |
| Private payment | 44236 (47.2) | 1881 (47.4) | 11054 (47.3) | 474 (45.1) |
| Other payment | 3139 (3.4) | 146 (3.7) | 731 (3.1) | 32 (3.0) |
| Self-payment | 2709 (2.9) | 68 (1.7) | 652 (2.8) | 17 (1.6) |
| Unknown | 150 (0.2) | 4 (0.1) | 33 (0.1) | 0 (0.0) |
| Race/Ethnicity |  |  |  |  |
| White | 21526 (23.0) | 927 (23.4) | 5298 (22.7) | 223 (21.2) |
| Black | 5870 (6.3) | 136 (3.4) | 1444 (6.2) | 45 (4.3) |
| Hispanic | 47123 (50.3) | 1888 (47.6) | 11797 (50.5) | 540 (51.4) |
| Asian | 14115 (15.1) | 801 (20.2) | 3522 (15.1) | 193 (18.4) |
| Other | 5011 (5.4) | 214 (5.4) | 1291 (5.5) | 50 (4.8) |
| Maternal education |  |  |  |  |
| <12 years | 17276 (18.5) | 721 (18.2) | 4298 (18.4) | 216 (20.6) |
| 12 years | 23112 (24.7) | 932 (23.5) | 5682 (24.3) | 244 (23.2) |
| >12 years | 48772 (52.1) | 2125 (53.6) | 12219 (52.3) | 529 (50.3) |
| Unknown | 19 (<0.1) | 0 (0.0) | 1153 (4.9) | 62 (5.9) |
| Maternal age |  |  |  |  |
| <18 years | 1678 (1.8) | 67 (1.7) | 403 (1.7) | 15 (1.4) |
| 18-34 years | 69023 (73.7) | 2922 (73.7) | 17286 (74.0) | 761 (72.4) |
| >34 years | 22925 (24.5) | 977 (24.6) | 5661 (24.2) | 275 (26.2) |
| Unknown | 19 (<0.1) | 0 (0.0) | 2 (<0.1) | 0 (0.0) |
| Any diabetes, yes | 15769 (16.8) | 746 (18.8) | 4034 (17.3) | 233 (22.2) |
| Any hypertension, yes | 19169 (20.5) | 777 (19.6) | 4773 (20.4) | 208 (19.8) |
| Perinatal smoking, yes | 3799 (4.1) | 123 (3.1) | 945 (4.1) | 38 (3.6) |
| Chorioamnionitis, yes | 1246 (1.3) | 82 (2.1) | 323 (1.4) | 16 (1.5) |
| Parity |  |  |  |  |
| Null | 31752 (33.9) | 1711 (43.1) | 7860 (33.7) | 421 (40.1) |
| 1 | 28545 (30.5) | 1065 (26.9) | 7132 (30.5) | 302 (28.7) |
| 2-4 | 30115 (32.2) | 1082 (27.3) | 7505 (32.1) | 296 (28.2) |
| ≥5 | 3145 (3.4) | 103 (2.6) | 830 (3.6) | 32 (3.0) |
| Unknown | 88 (0.1) | 5 (0.1) | 25 (0.1) | 0 (0.0) |
| Phototherapy, yes | 7908 (8.4) | 484 (12.2) | 1911 (8.2) | 135 (12.8) |
| Infant morbidity^§^, yes | 2255 (2.4) | 81 (2.0) | 584 (2.5) | 33 (3.1) |
| ^§^*Infant morbidity*, composite variable including bronchopulmonary dysplasia, necrotizing enterocolitis, respiratory distress syndrome, intraventricular hemorrhage, retinopathy of prematurity, patent ductus arteriosus, periventricular leukomalacia | | | | |
