# Supplementary material for: Predicting the risk of 7‐day readmission in late preterm infants in California: A population‐based cohort study
Source: Health Sci Rep. 2023 Jan 2;6(1):e994. doi: 10.1002/hsr2.994 (PMC9808150; doi:10.1002/hsr2.994)
Supplement: Supplementary file 3 — Supplementary information. [file HSR2-6-e994-s002.pdf]

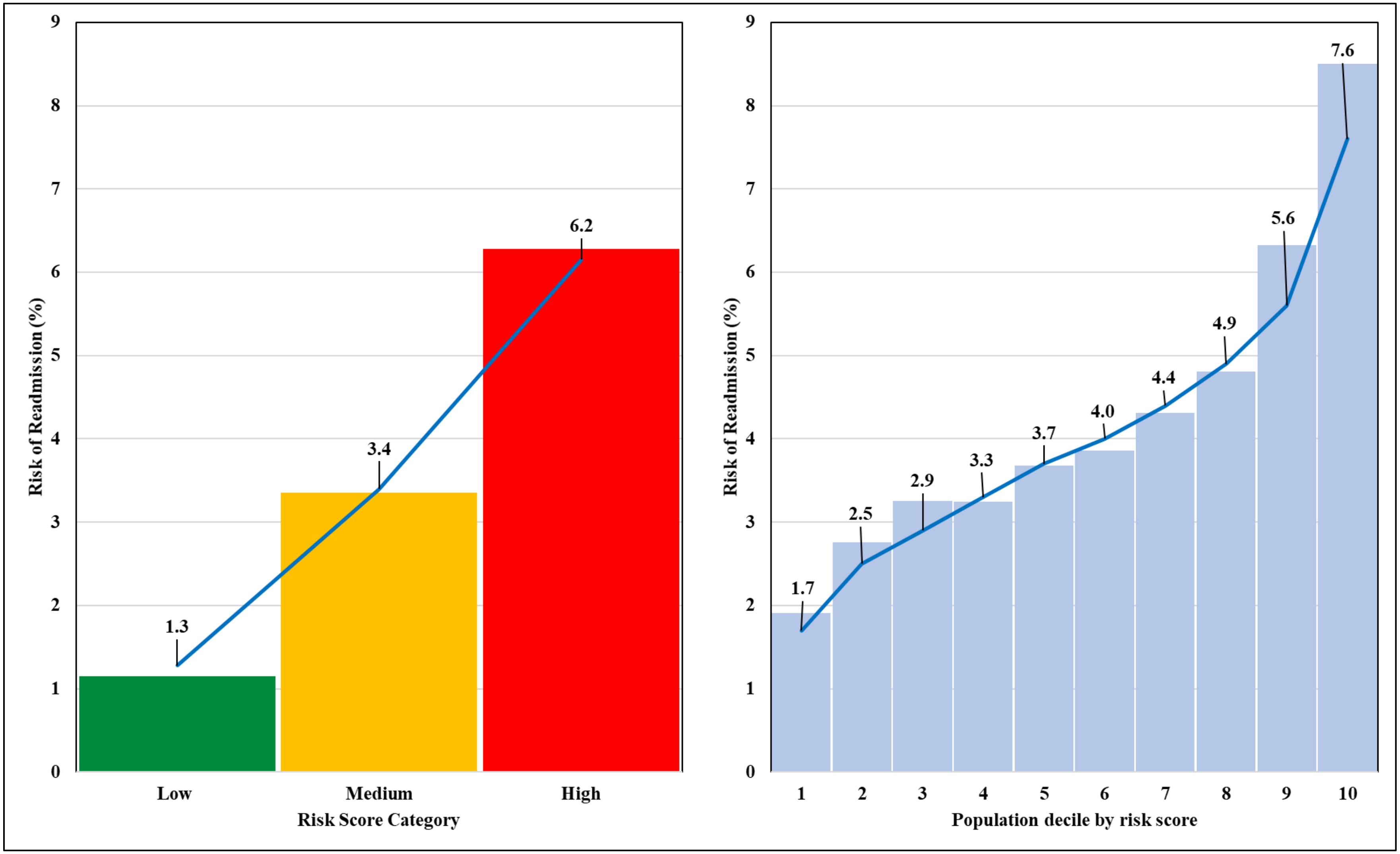

eFigure 1. Derivation sample caliberation plot in late preterm infants in California. Predicted (line) versus observed (bars) risk of readmission within 7-days for pre-defined risk categories and population deciles by risk score in the derivation sample (n=97,611).
Predicted risk (line) of readmission within seven days calculated by logistic regression mode
